# Flexible Electrodes as a Measuring System of Electrical Impedance Imaging

**DOI:** 10.3390/ma16051901

**Published:** 2023-02-24

**Authors:** Yi Wang, Xuecheng Ping, Xiaoyan Chen, Di Wang

**Affiliations:** 1 College of Mechanical Engineering, Tianjin University of Science and Technology, Tianjin 300222, China; 2 Tianjin Key Laboratory of Integrated Design and On-Line Monitoring for Light Industry & Food Machinery and Equipment, Tianjin 300222, China; 3 College of Electronic Information and Automation, Tianjin University of Science and Technology, Tianjin 300222, China

**Keywords:** electrical impedance tomography, flexible electronic technology, electrode, mechanical properties

## Abstract

Electrical Impedance Tomography (EIT) is a detection imaging technology developed 30 years ago. When the conventional EIT measurement system is used, the electrode and the excitation measurement terminal are connected with a long wire, which is easily affected by external interference, and the measurement result is unstable. In this paper, we developed a flexible electrode device based on flexible electronics technology, which can be softly attached to the skin surface for real-time physiological monitoring. The flexible equipment includes an excitation measuring circuit and electrode, which eliminates the adverse effects of connecting long wires and improves the effectiveness of measuring signals. At the same time, the design also uses flexible electronic technology to make the system structure achieve ultra-low modulus and high tensile strength so that the electronic equipment has soft mechanical properties. Experiments have shown that when the flexible electrode is deformed, its function is completely unaffected, the measurement results remain stable, and the static and fatigue performances are satisfactory. The flexible electrode has high system accuracy and good anti-interference.

## 1. Introduction

EIT is a non-invasive, radiation-free image reconstruction technique that has been extensively studied as a medical detection technology [1,2,3,4,5]. The basic work principle of EIT is to measure the response potential information by applying a safe driving current to the area to be measured in the human body and use the reconstruction algorithm to reconstruct the internal conductivity distribution image according to the different impedance characteristics of different tissues (or organs) or the same tissue (or organ) in different physiological and pathological periods [6,7,8,9]. EIT technology has the advantages of low cost, no radiation damage, and low requirements on the measurement environment, making it a research hotspot in the field of biomedical engineering [10,11,12,13]. The resistivity of human lung tissue is very different from most other soft tissues in the chest, and the resistivity of the lung will increase or decrease several times between inhalation and exhalation, so detecting the lung is currently the most promising clinical application of EIT [14,15,16]. The research results show that EIT technology is an effective monitoring technology for continuous monitoring and pulmonary disease screening [17,18,19]. When EIT technology detects the human body, multiple electrodes are required to be installed on the surface of the skin [20], and the electrodes are connected to the signal-processing module through long wires. However, when the electrode and the measurement system are connected by long wires, it is easy to cause adverse effects on the measured impedance, the measurement result is unstable, and the anti-interference ability is weak [21]. These adverse effects can be effectively eliminated by placing the excitation measurement circuit at the front end of the measurement system and directly connecting the electrodes, thus avoiding the long wire connection. However, the excitation measurement circuit itself is a rigid circuit, which can only be measured when the human body is sitting or standing upright. When the rigid circuit board is used for flat-lying, the experience of the user is extremely poor, and the circuit board is very easy to be damaged by the human body.

To solve the above problems, flexible circuits can be considered to replace traditional rigid circuits. Due to the excellent deformability of the flexible circuits, a series of novel applications have been developed, such as epidermal electronics for health monitoring [22,23,24], electronic skin [25,26,27], bionic electronic eyes [28,29], flexible displays [30], flexible sensors [31], and flexible batteries [32,33]. Flexible electronic technology is an emerging electronic technology that makes electronic devices on flexible substrates so that flexible electronic products can still work normally under complex deformation conditions, such as bending and stretching [34]. The functions of bending, folding, and stretching of flexible devices mainly rely on the excellent mechanical properties of flexible interconnecting wires. Therefore, the study of the mechanical properties of interconnecting wires has become the focus of academic research. Stretchability is an important index to evaluate the performance of flexible interconnection structures. In order to improve the stretchability of flexible interconnection, many scholars have conducted research on it. Zhou et al. [35] proposed a strain isolation layer design, pointing out that with the increase in the thickness of the base isolation layer during stretching, the serpentine interconnection transforms from local wrinkling to global buckling, which is helpful for the design of high-level elastic tensile capacity systems. Pan et al. [36] and Zhang et al. [37] studied the influence of the geometric parameters of the serpentine interconnection on the deformation mode and gave the wavelength of the buckling deformation of the straight line segment through theoretical analysis. Liu et al. [38] established a theoretical model for serpentine interconnection wires and studied the influence of geometric parameters, such as the length of straight line segments and arc radius, on stretchability. The above work has studied the influence of the parameters of the substrate or the interconnecting wire on the stretchability of the serpentine interconnection, which provides a guiding role for the improvement of the stretchability of the flexible interconnection. However, in the design of flexible electronic products, it is not only necessary to pay attention to the parameters of the substrate or wires but also to consider the overall structure of flexible electronics, the structure of serpentine interconnecting wires, and the combination mode of wires and substrates.

In this paper, flexible electronic technology is used to directly connect the excitation measurement circuit to the electrode to make a flexible electrode so as to shorten the transmission distance of weak AC signals and eliminate external interference. At the same time, it has basic physical properties that match human skin, such as appropriate thickness, low elastic modulus, and large elastic stretch level. We compared the flexible electrode with the traditional measurement system in terms of measurement effect, and the results show that the flexible electrode has higher system accuracy and better anti-interference performance, which improves the validity of the measurement signal. The flexible electrode fits perfectly with the human body, making it more convenient and comfortable to wear. At the same time, the deformation will not cause damage to the circuit.

## 2. Design and Experiment

### 2.1. Flexible Electrode Structure Design

**Figure 1:**
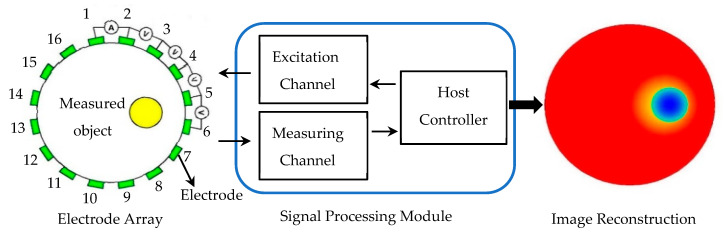
Electrical impedance tomography system structure.

**Figure 2:**
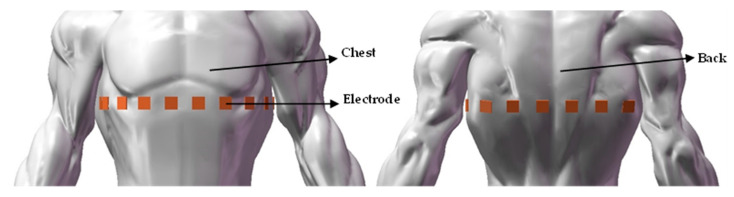
The electrode array is placed around the chest when the human lungs are tested.

The signal-processing part of the EIT system is composed of a measurement system and a control system, in which the measurement system directly affects the imaging results and is a key part of the EIT system [39]. Figure 1 shows a typical EIT system structure, which is mainly composed of three parts: an electrode array, a signal processing module, and an image reconstruction module. A total of 16 electrodes in the electrode array are evenly distributed on the surface of the measured object, and the electrical impedance of the yellow area is significantly different from other areas in the measured object. The signal processing module mainly includes a host controller, an excitation channel, and a measurement channel. The excitation channel is mainly responsible for the generation of the excitation source. The measurement channel is mainly responsible for collecting and processing measurement signals. The electrode pair (1,2) is used as the excitation electrode and the other electrode pairs (2,3), (3,4),…, and (16,1) as measurement electrodes. Then, the electrode pairs (2,3), (3,4),…, and (16,1) are respectively used as excitation electrodes to apply current signals, and the other electrode pairs measure voltage signals in the same way. After the obtained voltage signal is processed, the image is reconstructed by the image reconstruction algorithm. The image presents different colors according to the different impedance of the inner region of the measured object. The basic principle of this imaging is to obtain the impedance distribution data at a certain moment of measurement; the image reconstruction algorithm is used to obtain the absolute value of the impedance distribution in the field at this moment. Finally, the image reconstruction is realized by using the absolute value of the obtained impedance distribution. Figure 2 is the electrode array distribution model when testing the human lungs: 16 electrodes are evenly distributed around the chest of a human body.

**Figure 3:**
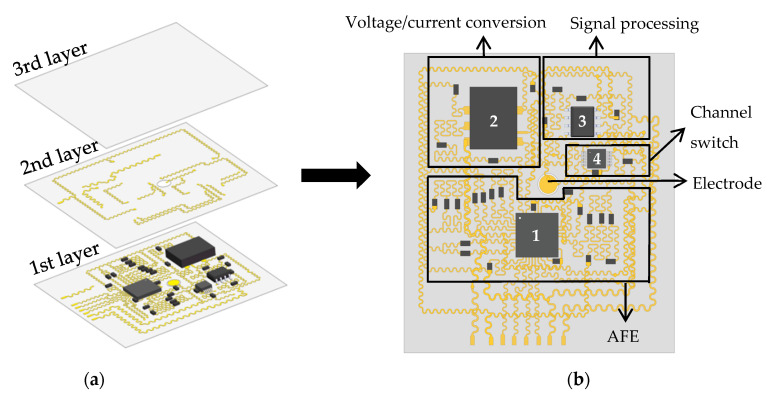
(**a**) Exploded schematic diagram of the various parts of the flexible electrode; (**b**) Assembled system schematic diagram, with wireframes and labels identifying the various subsystems.

Figure 3a is an exploded schematic diagram of various parts of the flexible electrode device capable of generating, converting, collecting, filtering, and amplifying electrical signals. The flexible electrodes are divided into a stretchable and bendable three-layer layout. The overall circuit is divided into two layers, the first layer is designed as an island-bridge structure, and the second layer is a separate serpentine copper wire structure. The circuit in the flexible electrode consists of thin and narrow serpentine copper wires with 200 um width and 100 um thickness and electronic components. These copper wires connect multiple chip-level integrated circuit components to each other, and the Ecoflex layer is used as the insulating layer between the two circuits. The flexible electrode is attached to the surface of the measured object, and the circular electrode is in contact with the object. The device uses the flexible Ecoflex as the substrate and encapsulation material; thus, the flexible electrode device as a whole can be stretched and bent. The flexible material Ecoflex uses Ecoflex 00–30 silicone rubber, which has a Shore hardness of 00–30.

The excitation measurement circuit mainly includes an analog front end (AFE), a signal processing module, a voltage/current conversion module, and a channel switch module, as shown in Figure 3b. Chip 1 (AD5941) is used as an AFE; its main function is to generate excitation signals and implement analog-to-digital conversion (ADC) and digital-to-analog converter (DAC). AD5941 uses an external 3.3 V power supply to generate a high-frequency sinusoidal voltage signal under the control of the host controller. Chip 2 (AD844) is used as a voltage-controlled current source (VCCS) to convert the voltage signal into a stable sinusoidal current signal with a peak value of 4.5 mA and a frequency of 100 kHz, which is output to the electrode. After the corresponding voltage signal is measured by other adjacent electrodes, chip 3 (LM6172) performs signal preprocessing so that the voltage value of the voltage signal is within the detection range of AD5941, and the analog signal is converted into a digital signal by ADC. Since each electrode can be used as an excitation electrode or a measurement electrode, but one electrode cannot have two states at the same time, the chip 4 (ADG1221) digital switch is selected as the channel switch to set the state of each electrode. The center of the circuit is a circular electrode, and eight wires extend out from the circuit to connect with the external circuit. Among them, four wires are signal wires, one wire is a ground wire, one wire is connected to an external 3.3 V power line, and two wires are connected to an external ±15 V power line.

**Figure 4:**
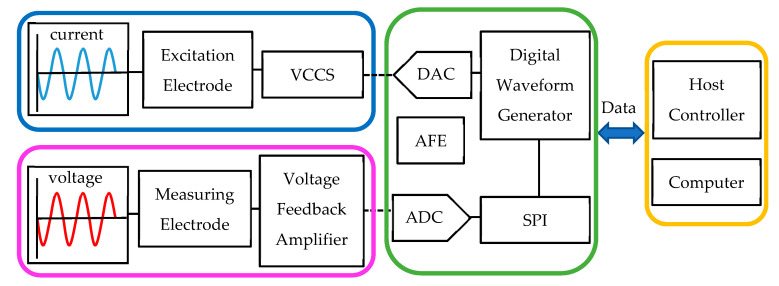
Simplified functional block diagram of the EIT.

The block diagram in Figure 4 briefly summarizes the system architecture of the EIT system we used. Each flexible electrode includes a circular electrode, a VCCS, a voltage feedback amplifier, and an AFE. Under the control of the host controller, the digital waveform generator generates a sinusoidal signal, which is first converted into an analog signal by a DAC, then converted into a current signal through the VCCS, and finally, output to the electrode. After the voltage signal is measured by measuring electrode pairs, the signal is preprocessed by the voltage feedback amplifier, and the analog signal is converted into a digital signal by the ADC and output to the computer for image reconstruction using the reconstruction algorithm.

**Figure 5:**
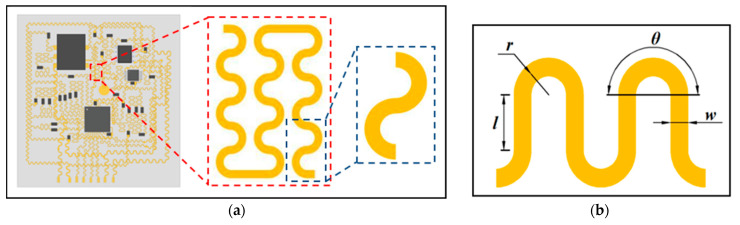
(**a**) Flexible electrode and an illustration of the “self-similar” serpentine wire geometry used for interconnection (the blue box is the first-level geometry, and the red box is the second-level geometry); (**b**) Four design parameters of serpentine wire.

The most widely studied structure in flexible electronics is the “island–bridge” structure, which uses stretchable interconnecting wires to connect rigid devices [40,41,42]. When external strain is applied, the interconnecting wires (bridges) have a lower effective stiffness and are prone to deformation in bending, which greatly reduces the strain on the rigid material (island) during stretching. For the design of interconnecting wires in an island-bridge structure, the concept of fractal design provides an efficient approach to simultaneously achieve large stretchability and high area coverage in stretchable electronics [43]. As shown in Figure 5a, the flexible electrode is designed as a rectangle of 45 mm × 48 mm, which provides an example of a fractal design in a flexible electrode as part of a second-order serpentine interconnect circuit. The fractal design introduces the serpentine configuration into the first-order structure and forms a second-order structure according to the self-similar geometry. Higher-order structures can also be easily designed and implemented, but considering the overall space layout and actual work conditions, the second-order structures can meet application requirements. Figure 5b shows the four design parameters of the serpentine wire, *θ* is the central angle of the serpentine wire, *r* is the arc radius of the serpentine wire, *w* is the width of the serpentine wire, and *l* is the length of the straight line in the serpentine wire.

When designing the flexible electrode, the numerical simulation of stretchability is carried out using the finite element software ABAQUS. The rubber material Ecoflex is selected as the flexible substrate. The material properties are set to hyperelasticity, and the Mooney–Rivlin model is used for the strain potential energy. This model describes the properties of the material with three parameters, *C*_10_, *C*_01_, and *D*_1_. The specific values are *C*_10_ = 0.008054, *C*_01_ = 0.00013, and *D*_1_ = 2, respectively. The metal interconnection material is copper with an elastic–plastic constitutive relation. Young’s modulus and Poisson’s ratio are set to 124 GPa and 0.34, respectively, and the yield strength is 372 MPa. A hexahedral (C3D8R) mesh is used in the mesh setup.

**Figure 6:**
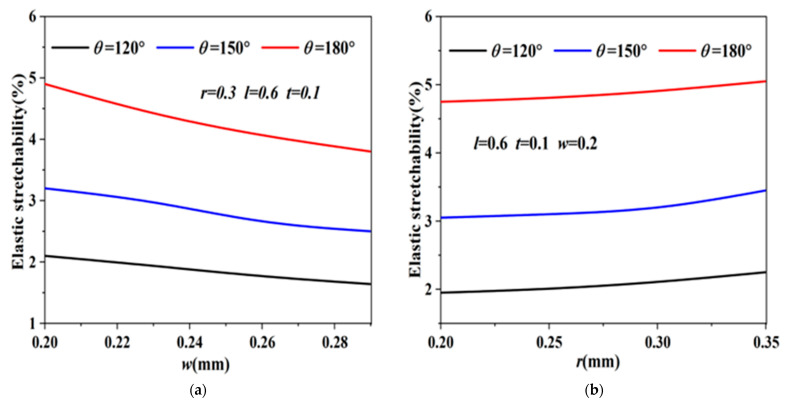

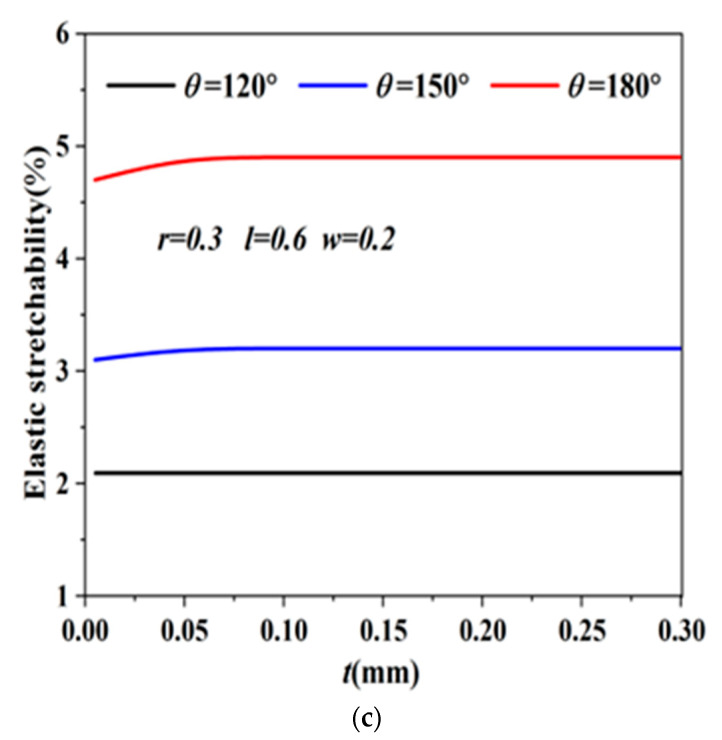
(**a**) The effect of the width *w* of the serpentine copper wire on the tensile properties when *θ* is 120°, 150°, and 180°, respectively; (**b**) The effect of arc radius *r* on the tensile properties when *θ* is 120°, 150°, and 180°, respectively; (**c**) The effect of copper wire thickness *t* on the tensile properties when *θ* is 120°, 150°, and 180°, respectively.

The tensile properties of the serpentine copper wire were simulated using the finite element method. Use *t* to represent the thickness of the serpentine copper wire. As shown in Figure 6a, under the fixed parameters of *r*, *l*, and *t* (*r* is 0.3 mm, *l* is 0.6 mm, *t* is 0.1 mm) and various central angle *θ*, the elastic stretch rate of the wire decreases significantly with increasing width *w* of the serpentine copper wire, so the width of the serpentine copper wire is selected as 0.2 mm. It can be seen from Figure 6b that when *θ* is 120°, 150°, and 180°, respectively, the elastic stretch rate of the serpentine copper wire increases with increasing *r*. In order to ensure that there is enough space between the wires, the arc radius *r* is selected to be 0.3 mm. It can be seen from Figure 6c that when the thickness *t* of the copper wire exceeds 0.1 mm, the elastic stretchability of the wire hardly changes with increasing wire thickness. Similarly, the wire can obtain the maximum tensile performance at *θ* = 180°, so the thickness of the serpentine copper wire is selected as 0.1 mm, and the radius of the arc *r* is taken as 180°. Because the overall circuit is complex and the serpentine wires are relatively dense, to ensure that the wires do not touch each other, the length *l* of the straight line segment in the wire is selected as 0.

**Figure 7:**
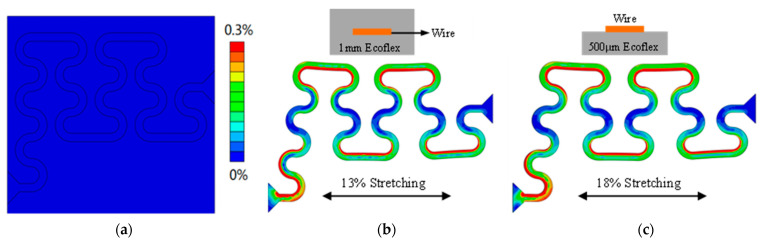
(**a**) The initial state of a section of interconnecting wire; (**b**) The interconnection wire is fully embedded in the middle of Ecoflex (Ecoflex thickness 1 mm) when the applied strain reaches its yield limit (13%), the strain distribution of the interconnection wire; (**c**) The interconnection wire is fully bonded on the top of Ecoflex (Ecoflex thickness 0.5 mm) when the applied strain reaches its yield limit (18%), the strain distribution of the interconnection wire.

Serpentine interconnects can be free-floating interconnects, fully bonded to the substrate, or fully embedded in flexible materials, and their mechanical behavior is qualitatively different [22]. Therefore, it is necessary to explore the effects of different combination methods of interconnection wire and substrate on stretchability. Free-floating interconnection means that there is no contact between the serpentine interconnection wire and the substrate. Usually, the electronic component is fixed on the substrate, and the free-floating serpentine interconnection wire and the electronic component are soldered together to indirectly constrain the mechanical behavior of the wire. Free-floating interconnects have few constraints and can generally have greater elastic stretchability. However, in more complex and dense circuits, the physical contact between interconnect wires cannot be well prevented when the device deforms, so free-floating interconnects are not used here. The finite element method is used to compare the case of interconnect wires bonded to the substrate with those embedded in the flexible material; Figure 7a is the initial state of a section of interconnection wire. The interconnection wires fully embedded in Ecoflex have an elastic stretching capacity of 13% at their yield limit, as shown in Figure 7b. While the interconnecting wires only bonded on the Ecoflex substrate, the elastic stretching capacity is 18% when reaching its yield limit, as shown in Figure 7c. Therefore, it is decided that the interconnection wires are bonded to the substrate in the present design.

### 2.2. Manufacturing Process of Flexible Electrodes

**Figure 8:**
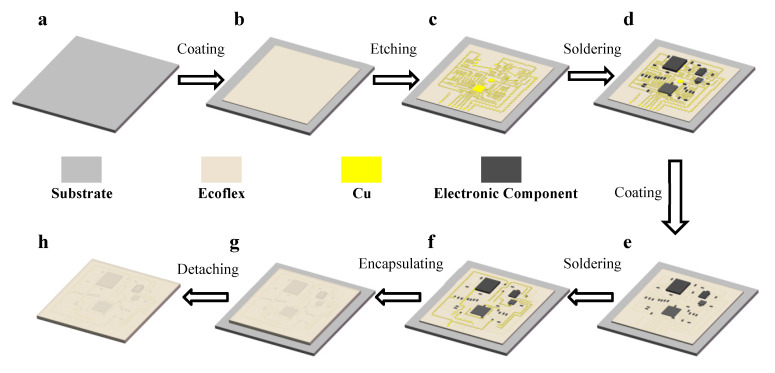
Schematic diagram of the fabrication process: (**a**) Glass substrate; (**b**) Ecoflex coated glass substrates; (**c**) Copper wires are fixed on Ecoflex; (**d**) Solder circuit components to copper wires; (**e**) Place a thin layer of Ecoflex coating insulation; (**f**) Solder the second layer of copper wires to the overall circuit; (**g**) Encapsulated with Ecoflex; (**h**) Flexible electrode detached from the substrate.

The processing method of the serpentine copper wire is an etching, and the copper foil (0.1 mm thick) is processed by photochemical etching according to the size parameters. The flexible electrode was fabricated starting from a flexible substrate coated with a 0.5 mm thick Ecoflex layer on a clean glass substrate (Figure 8a,b). Then, the prepared copper wires were fixed on it, and the circuit components were assembled and soldered with solder paste (Figure 8c,d). A new layer of Ecoflex coating was applied over the first circuit to insulate it from the second circuit (Figure 8e), and the second circuit was soldered into the overall circuit (Figure 8f). Finally, an Ecoflex layer was applied on top of the integral area of the circuit as an encapsulation layer; then, the device was detached from the glass slide (Figure 8g,h).

## 3. Results and Discussion

**Figure 9:**
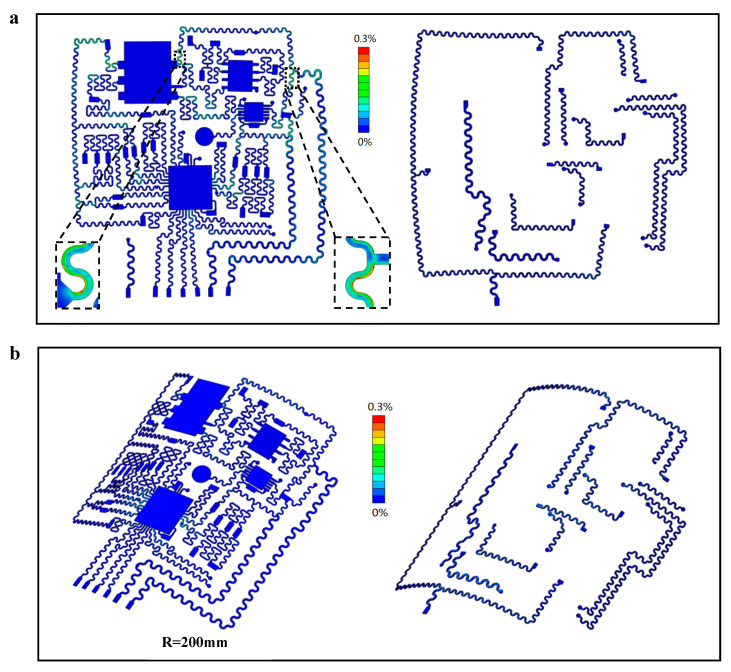
(**a**) Simulation results of the deformed geometry and strain distribution in the first (**left**) and second (**right**) copper layers of a flexible electrode during uniaxial tension (about 16%); (**b**) Finite element analysis results of copper layer deformation geometry and strain distribution for flexible electrodes at a bending radius of 200 mm.

Finite element analysis is used to study the mechanical properties of flexible electrodes, optimize the position of the chip and the structure of the serpentine interconnection, avoid the entanglement of the interconnection wires and the contact between the components, and improve the tensile performance at the same time. The purpose is to ensure that the equipment has certain tensile and bending performances. When deformed, the strain of the copper layer is below the elastic limit, and no plastic deformation occurs, so the deformation does not affect the use of the equipment. As shown in Figure 9a, the simulation results of the deformed geometry and strain distribution of the copper layer of the flexible electrode under uniaxial stretching in the horizontal direction show that the flexible circuit can be stretched by 16% without plastic deformation in the copper wire, which is much higher than the change of chest circumference when the human body takes a deep breath. Because the flexible electrode should fit the shape of the human body, it is required that the electrode is not only stretchable but also bendable. Therefore, a bending analysis was performed on the flexible electrode to fit it on a semicircular surface with a radius of 200 mm, and its deformed geometry and strain distribution were simulated by finite elements (Figure 9b). The results show that when the bending radius of the flexible electrode is smaller than that of the human body, the strain of the copper wire is far from reaching the yield limit. Therefore, according to the finite element analysis, the stretching or bending deformation of the flexible electrode during actual use will not cause damage to the circuit.

**Figure 10:**
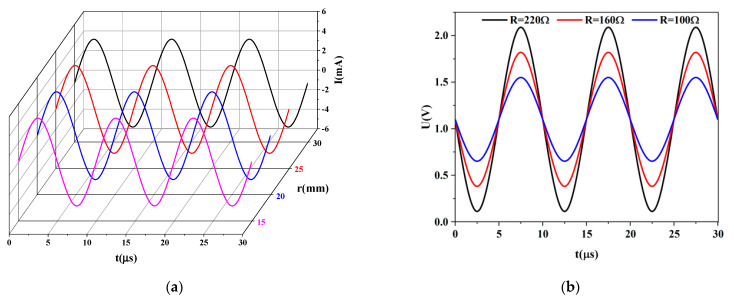
(**a**) The signal emitted by the excitation channel when the flexible electrode is attached to the cylindrical model with different curvature radii; (**b**) The signal output by the channel when measuring the resistance of different resistance values.

When this thin, stretchable electronic system is actually used, multiple flexible electrodes are evenly distributed around the area being measured. In order to ensure that the deformation of the circuit does not affect its function, the influence of circuit deformation on its function is verified through experiments. The main function of the overall circuit is to output analog current signals and output digital voltage signals after measurement. The flexible electrodes were attached to cylindrical models with radii of 30 mm, 25 mm, 20 mm, and 15 mm, respectively, and the analog signals output by them were measured. The host controller control circuit is set to output a sinusoidal current signal with a peak value of 4.5 mA and a frequency of 100 kHz. The experimental results show that the output signal of the flexible electrode does not change, which is the same as the setting parameters of the host controller, as shown in Figure 10a. Then, the digital voltage signal output after the flexible electrode measurement is tested. The electrodes are connected to resistors of different resistances, and their output voltage signals under a sinusoidal current with a peak value of 4.5 mA are obtained. The measured voltage signal is processed by dual high-speed voltage feedback amplifiers in the circuit. A negative bias voltage of 1.1 V is applied to the collected voltage signal; then, the phase of the signal is reversed after the bias voltage is applied by 180 degrees. Finally, the digital signal is output after being processed by the chip AD5941. In the experiment, the electrodes were connected to resistors with resistance values of 100 Ω, 160 Ω, and 220 Ω, respectively, and the measured digital voltage signals were consistent with the theoretical output results, as shown in Figure 10b. The above tests prove that when the flexible electrode undergoes large deformation, it will not affect the output and measurement results. The fatigue experiment with a uniaxial strain of 10% cyclic test was carried out on the flexible electrode at a frequency of 0.4 Hz using a ZQ-900 electric tension and compression testing machine. After 10,000 cycles of stretching, the device can still be used normally; it is proved that the fatigue resistance of the device meets the requirements.

The performance of the flexible electrode and the conventional electrode connecting the circuit with a long wire was evaluated by the two indexes: system accuracy and signal-to-noise ratio (SNR). A resistor with a resistance value of 10 kΩ is selected, and a sinusoidal current signal with a peak current of 0.2 mA, a frequency sweep range of 10~100 kHz, and a step size of 10 kHz is used for excitation. The average value was taken after 20 repeated measurements at each measurement frequency point. The relative error of the measured impedance of the flexible electrode and the conventional electrode at each frequency point is calculated via Equation (1), and the SNR at each frequency point is calculated according to Equation (2).

(1) $$E=\frac{\left|\frac{1}{n}\sum_{i=1}^{n}x_{i}-A\right|}{A}\times 100\%,$$

(2) $$\mathrm{SNR}=-20\mathrm{lg}\left(\sqrt{\frac{1}{n-1}\overset{n}{\underset{i=1}{\sum }}{\left(x_{i}-\frac{1}{n}\overset{n}{\underset{i=1}{\sum }}x_{i}\right)}^{2}}/\left(\frac{1}{n}\overset{n}{\underset{i=1}{\sum }}x_{i}\right)\right)$$

where $x_{i}$ is the measured impedance measurement value at the i-th frequency, *A* is the actual value of the measured impedance, *n* is the number of measurements, *E* is the impedance error, and SNR is the signal-to-noise ratio.

**Figure 11:**
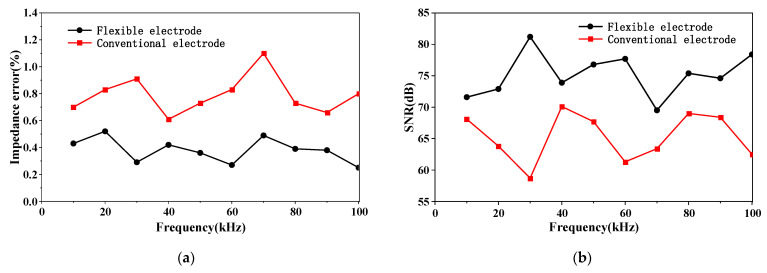
(**a**) Impedance error at each frequency; (**b**) SNR at each frequency.

Draw the curve according to the calculation results of Equations (1) and (2), as shown in Figure 11. The figure shows that the impedance error measured by the flexible electrode at each frequency point is within 0.6%, the average impedance error is 0.38%; the SNR is greater than 70 dB, and the average SNR is 75.2 dB. The impedance error of the conventional electrode at each frequency point is less than 1.2%, and the average impedance error is 0.78%; the SNR is greater than 55 dB, and the average SNR is 65.3 dB. It shows that the flexible electrode has higher system accuracy and better anti-interference than the conventional electrode.

**Figure 12:**
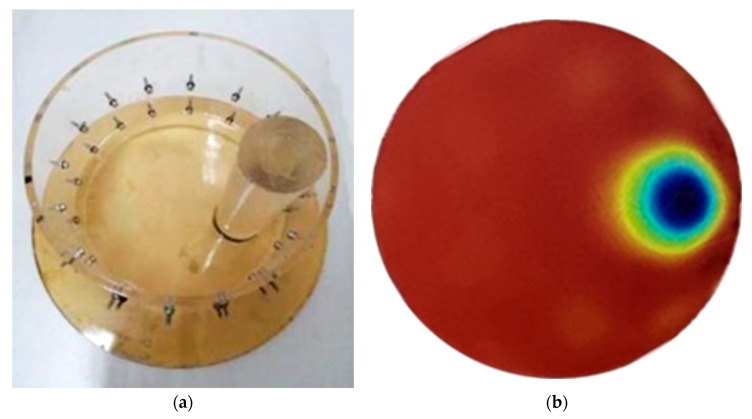
(**a**) Salt water tank for organic glass rod; (**b**) EIT imaging result.

Flexible electrodes are used to test real objects. An organic glass rod is placed in the brine tank with a diameter of 20 cm, and brine is injected for EIT imaging, as shown in Figure 12a. Figure 12b is an EIT imaging result. It is not difficult to see from the image that the EIT system can identify and locate the organic glass rod, and the size, shape, and position of the organic glass rod in the imaging figure are basically consistent with the actual situation. Therefore, flexible electrodes also perform well in practical use.

## 4. Conclusions

In this paper, flexible electrodes that are different from those used in traditional EIT techniques are designed. The excitation measurement circuit is integrated into the electrode to make a flexible integrated circuit, which shortens the transmission distance of the weak AC signal and improves the effectiveness of the measurement signal. The following conclusions are drawn through simulation and experiments as follows:

(1) Serpentine copper wires are used to connect electronic components, and flexible materials are used for packaging and insulating the circuits. This design realizes a system structure with ultra-low modulus and high tensile properties; satisfactory tensile and bending properties and fatigue resistance are obtained, which meet the requirements for practical use.
(2) Experiments on the excitation and measurement functions of flexible electrodes prove that when the device is deformed, the excitation and measured signal of the flexible electrode will not change. Moreover, the experimental data show that flexible electrodes have higher system accuracy and better anti-interference performance than conventional electrodes.

Present results demonstrate the use of flexible electronics to combine circuits with electrodes, and the result is a thin and comfortably flexible electrode device that fits softly on the surface of the skin for real-time physiological monitoring. For the digital multi-electrode EIT system, how to reduce the system complexity and optimize the electrode design will become our further research content.

## Data Availability

Not applicable.
